# Therapeutic Remodeling of the Gut Microbiome as a Strategy to Restore Immune Tolerance in Autoimmunity

**DOI:** 10.1002/mbo3.70294

**Published:** 2026-04-19

**Authors:** Behshad Boroumand, Amirhossein Jaberi, Ghazal Zamani, Ehsan Zandi, Farshad Zare, Milad Vahedinezhad, Elham Abdollahi, Sepideh KarkonShayan, Sasan GhazanfarAhari, Mustafa Sattar

**Affiliations:** ^1^ Pharmacology and Toxicology Department Shahid Sadoughi University of Medical Sciences and Health Services Yazd Iran; ^2^ Medical School Shiraz University of Medical Science Shiraz Iran; ^3^ Student Research Committe, Shiraz University of Medical Sciences Shiraz Iran; ^4^ School of Medicine Shiraz University of Medical Sciences Shiraz Iran; ^5^ Student Research Committee, School of Medicine Tabriz University of Medical Sciences Tabriz Iran; ^6^ Cardiovascular Research Center, Faculty of Medicine Tabriz University of Medical Sciences Tabriz Iran; ^7^ Faculty of Medicine Tabriz University of Medicine Sciences Tabriz Iran

**Keywords:** autoimmune diseases, dysbiosis, gut microbiome, immune modulation, probiotics

## Abstract

Autoimmune diseases happen when the immune system, which is supposed to defend the body from infections and other harmful things, starts to attack the body's own cells by mistake. In the last few years, they seem to be getting more public, and the reasons are quite complicated. It is usually not just one factor, but a mix of genes and environmental influences, such as diet, infections, or even stress. The gut microbiome, the vast community of bacteria and other tiny organisms living in our intestines, plays an important role in shaping how the immune system behaves. When this gut microbiota becomes unstable (a state called dysbiosis), it can be associated with the onset or worsening of various autoimmune diseases. In this review, we discuss the close relationship between the gut microbiome and autoimmune disorders and focus on how the microbiome can affect immune activation, immune tolerance, and inflammation at the molecular level. The general idea is that, if we understand these interactions better, we might be able in the future to design new ways to manage autoimmune diseases earlier and maybe in a more personalized way. In the end, the review suggests that if we understand better how the microbiome is involved in autoimmune diseases, it might be possible in the future to design more personalized therapies that change gut bacteria in a smart way and hopefully improve patient outcomes.

## Graphical abstract

This graphical abstract illustrates how altered gut microbiota lead to autoimmunity. It shows that gut dysbiosis causes a “leaky gut” and activates autoreactive intestinal immune cells, which then migrate to other organs, ultimately triggering systemic autoimmune responses.

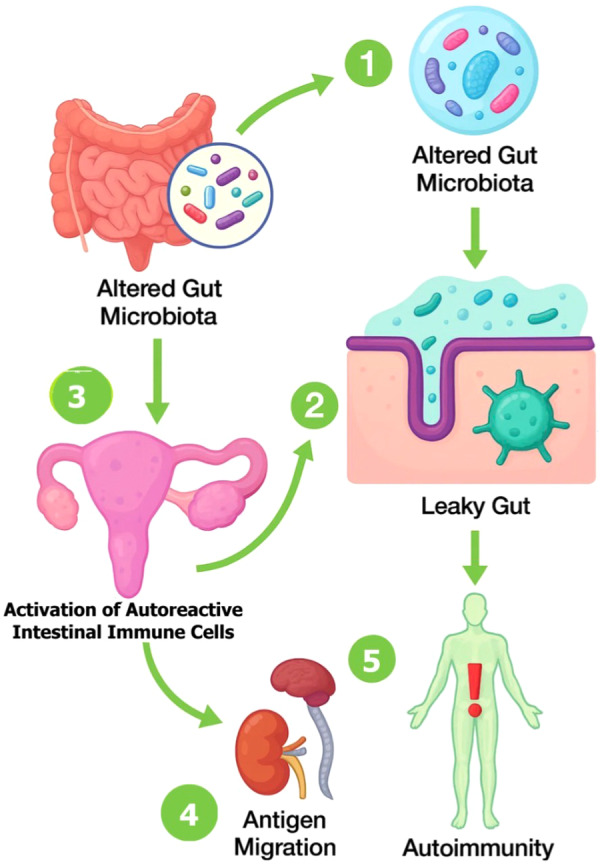

**Abbreviations**
AIDs: autoimmune and autoinflammatory disorders
APC: antigen‐presenting cell
FMT: fecal microbiota transplantation
GALT: gut‐associated lymphoid tissue
GI: gastrointestinal
HLA: human leukocyte antigen
HLA‐B27: human leukocyte antigen B27
IBD: inflammatory bowel disease
IFNα: interferon alpha
IL‐17: interleukin‐17
IL‐1β: interleukin‐1 beta
IL‐6: interleukin‐6
mRNA: messenger RNA
MS: multiple sclerosis
RA: rheumatoid arthritis
SCFAs: short‐chain fatty acids
SLE: systemic lupus erythematosus
TCR: T‐cell receptor
Th17: T‐helper 17 cell
TLR(s): toll‐like receptor(s)
Treg(s): regulatory T cell(s)

## 1 Introduction

Autoimmune diseases happen when the immune system, which normally should protect the body from infections and other outside things, starts to attack the body's own tissues by mistake. This strange reaction usually does not come from just one cause, but from a mix of genetic background, environment, and also changes in the gut microbiota (Omar et al. 2025; Karimi et al. 2024). The gut microbiota is a big community of different microorganisms, like bacteria, viruses, sfungi and even protozoa, that normally live together in balance in our digestive system. In a normal situation, the immune system in the gut tries to tolerate or neutralize most of the antigens that come from food or microbes. But when this balance is disrupted (dysbiosis) or the mucosal barrier is damaged, harmful antigens can reach immune cells more readily, triggering inflammation beyond what is necessary. If this inflammation continues for a long time, it can turn into a systemic immune response and finally contribute to autoimmune disease development (Tokuhara et al. 2019; Yacoub et al. 2018).

In this review, we look at the complex relationship between the gut microbiome and autoimmune diseases, and we summarize experimental data that show how changes in microbiota composition are linked to autoimmune conditions, in both human and animal studies (Gyriki et al. 2025). One important immune cell type in this story is T‐helper 17 (Th17) cells, which seem to increase in many autoimmune diseases, and their levels often go together with disease severity. We also discuss how vaccine‐induced immune responses might interact with the microbiome and maybe help reduce or control some autoimmune diseases. Because of all this, the gut microbiome might become a kind of diagnostic marker in the future, for example, to predict which patients are at higher risk or how fast a disease will progress (L. Zhang, Qing, et al. 2021). Autoimmune and autoinflammatory disorders (AIDs) are now a big global health problem. The microbiome, which includes not only microbes but also the way they interact with host genes and environment, has a key role in shaping immune responses. Things like the gut–brain axis and genetic predisposition are some of the links between the microbiome and these disorders. However, if interest in microbiome research is growing a lot, studies that focus on single diseases often give mixed or even opposite results (Carucci et al. 2021). Because of that, we need a more holistic view that considers genetics, clinical features, and microbiome changes together to better understand how they are connected. Systematic reviews can show how the microbiome differs across different autoimmune diseases and identify common patterns in microbial diversity. This can improve our understanding of how the microbiome influences immune responses and disease mechanisms (Scher et al. 2025). In this review, we bring together recent findings and try to give an overview of the impact of the microbiome on autoimmune diseases, using concepts like alpha and beta diversity to describe these changes in a more structured way.

The main goal of this review is to make a clearer link between autoimmune diseases and factors, such as antibiotic use and eating habits, especially foods that can modulate the immune system, like fibers and artificial or natural sweeteners (Posta et al. 2023). By collecting and comparing current studies on the microbiome in autoimmune diseases, we try to find trends and shared connections between different conditions. Another important aim is to suggest a basic framework for future research, so that new studies can be compared more easily and the field can move a bit more in the same direction (García‐Velasco et al. 2020). We also point out that there are still many differences and even contradictions in published results, which show the need for more careful, well‐designed studies if we want a clearer picture of how the microbiome affects the onset and progression of autoimmune diseases. In addition, we discuss what it might mean to “manipulate” the microbiome, for example, to prevent or treat both new and already existing diseases (Yuan et al. 2025). This includes looking at problems in current methods, the limited number of strong clinical trials, and the fact that there are still not many solid treatment strategies based directly on microbiome data (Supianto 2025). In the end, we argue that interdisciplinary work, bringing together immunology, microbiology, genetics, bioinformatics, and clinical medicine, is really needed to better understand how the microbiome contributes to autoimmune disease and how we might use this knowledge for new therapeutic approaches.

## 2 Understanding the Microbiome

The human microbiome, which means all the trillions of tiny organisms that live with us in and on our bodies, is now seen as a very important part of our health and immune system. The diversity of this microbiome helps the body to fight against pathogens, control inflammation, and keep the immune response more or less balanced. But when this balance is disturbed, for example, by antibiotics, strong stress, infections, or big changes in diet, the microbiome can shift in a bad way and may increase the risk of autoimmune diseases (Singh and Bhadauriya 2025). Studying the microbiome is, therefore, really important if we want to understand how it is connected to immune diseases and how we might use it as a target for therapy. Many studies are trying to see how microbial communities “talk” to the immune system and how this can either protect us or, in some cases, promote disease. With a better and deeper understanding of these processes, it should be possible to design new strategies for managing autoimmune diseases and similar conditions, hopefully leading to better outcomes for patients (Singh and Bhadauriya 2025; Mkilima 2025).

In animal models, gut bacteria have shown they can change autoimmune responses, for example, in experimental models of neurological autoimmune diseases, thyroiditis, or gastritis. Early colonization of the gut with beneficial microbes in infancy seems especially important for the development of a healthy immune system and may influence a person's likelihood of developing an autoimmune disease later in life (Addissouky et al. 2025). A balanced gut microbiome is also essential for controlling chronic inflammation, and when this balance is lost, it can make AIDs worse. High levels of proinflammatory metabolites in the gut or oral microbiome can weaken the gut barrier and allow harmful substances like lipopolysaccharides and autoantigens to pass through more easily, which then activates the immune system and supports the persistence of autoimmune disease (Gioitta Iachino et al. 2024).

The gastrointestinal (GI) microbiome (or microbiota) is usually described as a collection of bacterial communities in the gut. But in reality, it also includes other microbes, such as fungi and viruses, for example, in the biliary microbiome that is linked with some liver diseases. Overall, the microbiome is incredibly diverse, with thousands of different species of bacteria, viruses, fungi, and even archaea living together. New “omics” technologies, like metatranscriptomics and metaproteomics, have shown that what the microbiome is doing (its function) does not always match exactly with which species are present (its taxonomy). This means that the role of a certain microbe can sometimes be more important than just knowing its name or group (Armengaud 2025; Athanasopoulou et al. 2023).

The human gut can contain around 10^11^–10^12^ bacteria per gram of feces, which creates a huge amount of genetic diversity that is much higher than the number of genes in the human genome itself. Some estimates say that the microbial gene pool can be up to 150 times bigger than the host's genome, which really shows how important these microorganisms are for our biology and daily body functions (W. Huang, Yin, et al. 2021; Yin et al. 2024).

The development of the immune system is shaped by both genes and environmental influences, and the gut microbiota has a key role in this maturation process. More and more research shows that immune health is closely linked to the composition of the microbiota. The early colonization of the gut with beneficial microbes after birth is critical not only for defense against infections but also for preventing chronic inflammatory conditions later on. Twin studies, for example, have shown that people who share a more similar microbiota also tend to have more similar immune profiles, which underlines how important microbiota diversity is for immune system development (Marquez‐Paradas et al. 2025; Schoultz et al. 2025).

Overall, the microbiome has strong effects on the immune system: it helps decide how well the body can defend itself, how immune tolerance is set, and how the body reacts to harmful pathogens. The balance of microbes in the gut—and also in other places like the lungs or the skin—has a big impact on how different immune cells work, including both the innate and adaptive branches. It also affects how immune tolerance is built at local epithelial surfaces and in the whole body, shaping the profiles of immune cells that are responsible for defense and tissue repair. Because of this, the microbiota is essential for keeping immune homeostasis and for preventing the immune system from attacking the body's own tissues, which would lead to autoimmunity (Iweala and Nagler 2019; Shao et al. 2023).

## 3 Immune Dysregulation and Its Consequences

Autoimmune diseases are a growing group of long‐lasting illnesses where the immune system, instead of only fighting viruses and bacteria, starts to attack the body's own cells and tissues by mistake. In these conditions, the normal self‐tolerance of the body breaks down, so the adaptive immune system becomes kind of “confused” and turns against the host. This usually shows up as self‐reactive T cells and autoantibodies in the blood that target specific organs or sometimes the whole body, which slowly leads to inflammation and tissue damage over time (Ahsan 2017; Y. Zhou and Jiang 2023). Genes are important for determining who is at higher risk of autoimmunity, but genetics alone is not enough. They work together with different environmental factors. Among these, the gut microbiota has become one of the main areas of focus (Lehman et al. 2023). The word “microbiota” means the full community of microorganisms living in and on the human body, bacteria, fungi, archaea, protozoa, viruses, plus all of their genetic material. These microbes are in close contact with immune cells all the time, and there is a two‐way interaction between them. This relationship can help keep the immune system calm and balanced, or if something goes wrong, it can push the system toward disease (Rojas et al. 2022).

Normally, the microbiota helps with immune regulation, supports tolerance, and stops the immune response from being overactivated all the time. But under some conditions, for example, after strong antibiotics, infections, or diet changes, the microbiota can shift into a more proinflammatory state. In that case, it may contribute to the start and progression of autoimmune and inflammatory diseases (Awuah et al. 2023). Because this relationship is so complicated, keeping a more or less balanced microbiome seems very important for general health. Experimental work with humanized animal models has shown that early exposure to a wide variety of microbes is important for building proper immune tolerance (Aminu et al. 2024). On the other hand, very “clean” environments with limited microbial exposure, sometimes called the hygiene hypothesis, can lead to immune dysregulation and may speed up the development of autoimmune diseases like type 1 diabetes. Interestingly, some environmental microbes might actually be protective, acting like natural trainers for the immune system that support homeostasis and help prevent chronic immune‐mediated disorders (Aboulaghras et al. 2024).

## 4 Origins and Triggers of Autoimmune Reactions

The start of autoimmune diseases is usually linked to a slow loss of self‐tolerance, when immune cells begin to see the body's own molecules as if they are something foreign. Step by step, this can lead to tissue damage, mainly through T‐cell‐mediated attacks. A clear example is type 1 diabetes, where immune cells attack and destroy the insulin‐producing beta cells in the pancreas (Gearty et al. 2022). In many patients, the disease process begins in people who already have a genetic risk, and then the immune system becomes sensitive to self‐antigens after some environmental or microbial trigger. When autoreactive T cells are not fully removed in the thymus, they can later be reactivated in peripheral tissues by things like infections or other stimuli. This reactivation starts a chain of immune reactions that, after some time, shows up as clinical disease (Kawakami and Wekerle 2024). With each round of activation and tissue injury, the autoimmune process gets stronger and more chronic.

Both genes and environment work together in this. Infections, diet, and changes in the microbiome can all influence how and when autoimmunity appears. In some rare situations, autoimmune features can even be transferred by hematopoietic stem cell transplantation from a donor who already has an autoimmune disease, which shows that immune dysfunction can stay “built in” to certain immune cell lineages (Müskens et al. 2021). This supports the idea that self‐reactive immune cells can stay quiet for a long time and then become active again when peripheral immune control is disturbed. The thymus is very important for educating T cells and creating self‐tolerance during early life, but its exact role in controlling autoimmunity in adults is still not completely clear. Overall, the development of autoimmune disease is the result of a complicated mix of inherited susceptibility, environmental exposures, and the way the immune system interacts with microbial and metabolic signals in the body (Vitozzi et al. 2024; P. Zhang and Lu 2018) (Table 1).

**Table 1:** Key aspects of immune response in autoimmunity.

Key aspect	Description	Impact on autoimmunity	Key factors	Examples	
Gut microbiome	Dysbiosis of gut microbiota plays a key role in autoimmune diseases by disrupting immune tolerance.	Disrupted microbiota contribute to immune activation and inflammation, promoting autoimmune diseases.	Gut microbiota imbalance, infections, diet, and environmental factors.	Changes in gut microbiota composition linked to autoimmune diseases.	Paul et al. (2025) and Pelc et al. (2025)
Immune system regulation	The immune system mistakenly attacks the body's own tissues due to an altered microbiome.	The immune system attacks healthy cells inappropriately, contributing to disease development.	Alterations in gut microbiota impact immune tolerance, leading to inflammation.	Immune dysregulation in diseases like multiple sclerosis, rheumatoid arthritis.	Omar et al. (2025) and Zaroon et al. (2024)
T‐helper 17 cells	T‐helper 17 cells, a critical immune cell population, correlate with disease severity in autoimmune conditions.	Elevated Th17 cells contribute to the pathology of conditions, such as RA, MS, and Sjögren's syndrome.	T‐helper 17 cells are a marker for autoimmune disease severity.	Elevated Th17 cells in autoimmune diseases, like MS and RA.	S. Li et al. (2017) and Ulges et al. (2016)
Cytokine imbalance	Proinflammatory cytokines are increased, while anti‐inflammatory cytokines are reduced due to microbiota imbalance.	The imbalance in cytokine production exacerbates the immune response and drives tissue damage.	Environmental triggers like infections and diet influence cytokine levels.	Cytokine imbalances lead to chronic inflammation in autoimmune diseases.	Gutiérrez‐Salmerón et al. (2021) and Leblhuber et al. (2021)
Microbiome and autoimmunity	Microbial alterations in the gut influence autoimmune disease onset, exacerbating systemic inflammation.	Microbial alterations in the gut influence autoimmune disease onset, exacerbating systemic inflammation.	Dysbiosis of gut microbiota and its interactions with immune cells influence autoimmune disease development.	Gut microbiota modulates immune responses in conditions, such as lupus and celiac disease.	Krishnareddy (2019) and Rahbar Saadat et al. (2019)

Abbreviations: MS, multiple sclerosis; RA, rheumatoid arthritis; Th17, T‐helper 17 cell.

## 5 Immune Activation and Its Implications in Autoimmunity

Dysregulation of immune pathways is basically the main way that problems in the gut microbiota can start or make autoimmune diseases worse. The gut‐associated lymphoid tissue, which has a lot of immune cells like dendritic cells, macrophages, and innate lymphocytes, is a really important part of the body's defense system. When the gut microbiota goes out of balance, it can wrongly activate these immune pathways and this often leads to more proinflammatory cytokines and less anti‐inflammatory ones, so the situation moves more toward chronic inflammation and autoimmune disease progression (Candelli et al. 2021; Lauriano et al. 2023).

**Figure 1:**
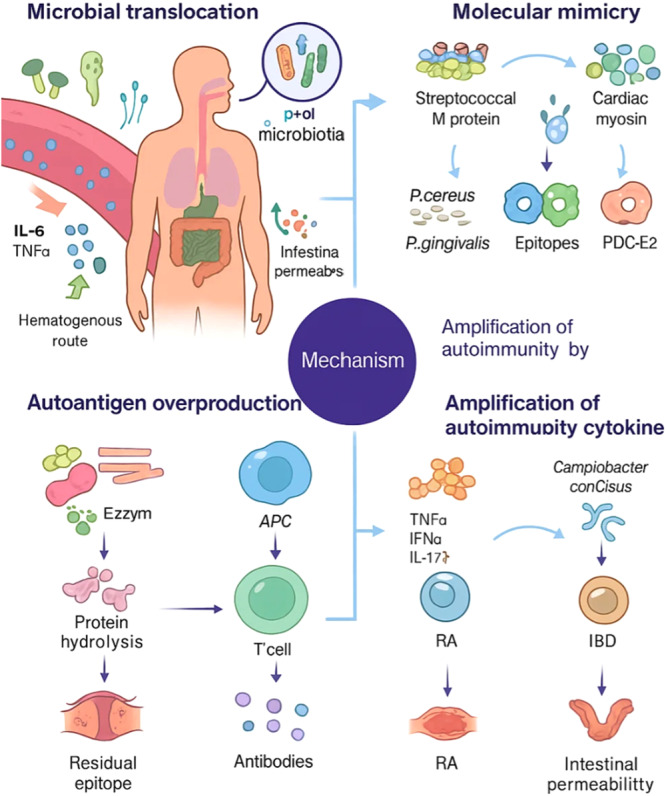
This figure shows how oral microorganisms can play an important role in autoimmune diseases through a few main pathways, like microbiota translocation, molecular mimicry, over‐production of autoantigens, and cytokine‐driven autoimmunity. In the top left part, it shows that the oral microbiota can communicate with the host through the blood (hematogenous route) and also through the gut (enteral route). When there are harmful conditions in the mouth, for example, ulcers or periodontal damage, oral pathogens such as *Porphyromonas gingivalis*, *Streptococcus mutans*, and other periodontal bacteria can move to distant organs. This movement can increase cytokine release and also affect the integrity of the intestinal barrier. Changes in the oral microbiota composition may also make molecular mimicry stronger, increase autoantigen production, and support cytokine‐mediated amplification of autoimmunity. In the top right part of the figure, it shows that antigenic epitopes from bacteria like *Treponema denticola*, *Bacillus cereus*, *P. gingivalis*, *Fusobacterium nucleatum*, and different *Streptococcus* species are increased. In the bottom left, the picture explains that microbial enzymes do hydrolysis on proteins, and this makes more residual and citrullinated epitopes. These leftover pieces are then taken up by APCs and shown to T cells, which starts inflammatory responses. This T‐cell activation then turns on B cells too, so they begin to make antibodies. In the bottom right part, pathogenic microbes such as *P. gingivalis*, *F. nucleatum*, and *Campylobacter concisus* stimulate the release of many proinflammatory cytokines, including TH1/TH17 cytokines, TNF‐α, IFNα, IL‐1β, IL‐6, and IL‐17. The figure also uses some short forms: P.g means *P. gingivalis*, k.s stands for *Klebsiella* strains, c.c is *C. concisus*, APC is antigen‐presenting cell, TLR is Toll‐like receptor, RA is rheumatoid arthritis, IBD is inflammatory bowel disease, Ro60 is related to anti‐dsDNA antibodies, and PDC‐E2 refers to the pyruvate dehydrogenase complex E2. IBD, inflammatory bowel disease; IFNα, interferon alpha; IL, interleukin; RA, rheumatoid arthritis; TNF‐α, tumor necrosis factor‐alpha.

One important mechanism in many autoimmune diseases is the dysfunction of T‐helper 17 (Th17) cells and their main cytokine, interleukin‐17 (IL‐17). This pathway is especially involved in diseases like rheumatoid arthritis (RA), Sjögren's syndrome, and multiple sclerosis (MS), where it adds to local inflammation and tissue damage. In type 1 diabetes, for example, cytokines such as TNF‐α, IL‐1β, and IL‐6 play a key role in killing the insulin‐producing beta‐cells, so they push the disease forward. In a similar way, autoreactive T cells do not only attack pancreatic beta‐cells in type 1 diabetes, but can also target intestinal epithelial cells in celiac disease, which causes more organ damage and speeds up disease progression (J. Huang, Xu, et al. 2021; Vasilev et al. 2025). Activation of pattern‐recognition receptors like toll‐like receptors on immune cells also has an important part in the development of autoimmune conditions, such as systemic lupus erythematosus (SLE). Studies have shown that dysbiosis in the gut microbiota of SLE patients can promote lymphocyte activation and increase the differentiation of Th17 cells, which makes the autoimmune reaction even stronger. On top of that, specific changes in the gut microbiota, for example, an increase in some bacterial species, have been linked with worse symptoms in different autoimmune diseases. These findings give new ideas about how the gut microbiota might influence disease activity and progression over time (D. Li et al. 2021; Quintero‐González et al. 2022; Wen et al. 2023) (Figure 1).

## 6 The Relationship Between Microbiome and Autoimmune Disorders

### 6.1 Direct Immune–Microbiome Interactions

The microbiome talks directly with the host immune system, especially when pathogens or different kinds of stress (like infection or strong drugs) trigger immune reactions. One example is the immunosuppressive effect of lactate that is made by *Lactobacillus* during its growth. Also, small microbial pieces, like peptides from bacteria, viruses, fungi, and protozoa, can cause cross‐reactive immune responses, which in some cases may end up in autoimmunity (Dubik et al. 2022; Shinde and Deokar 2024). This shows how delicate the balance is between the immune system tolerating normal commensal microbes and, at the same time, stopping harmful immune responses. Microbes also shape immune responses through epigenetic changes and by making short‐chain fatty acids (SCFAs), which can change immune signaling and help control inflammation (McCrory et al. 2024; L. Zhang et al. 2023).

Some species, for example, Eubacterium limosum, can change immune cell populations by influencing the balance between T‐helper cells and regulatory T cells. When these bacteria are present in the gut at the right time and amount, they can support the expansion of regulatory T cells, which help keep immune tolerance and prevent autoimmune reactions. But in some situations, like when colonization happens later in life or in an already inflamed gut, the same microbes might instead drive more proinflammatory responses and contribute to autoimmune disease development (Pavlova 2024; Sprouse et al. 2019).

### 6.2 Indirect Impacts of Dysbiosis on Autoimmune Disease

New research increasingly shows that gut dysbiosis and disturbed oral microbiota can contribute to the development of autoimmune diseases. Because the microbiome can affect both innate and adaptive immune responses, any disturbance in the microbial balance can indirectly change immune homeostasis (Jahani‐Sherafat et al. 2023; Khor et al. 2021). For example, when gut bacteria are out of balance, intestinal permeability can increase. This so‐called “leaky gut” lets microbial products pass into the blood and activate the immune system, which leads to systemic inflammation. Over time, this kind of situation can promote proinflammatory pathways and break immune tolerance, and this has been linked to several autoimmune diseases (Seton and Carding 2025).

Some autoimmune conditions may also be affected by microbes coming from the mouth. *Porphyromonas gingivalis*, a well‐known periodontal pathogen, has been associated with RA because it can produce citrullinated proteins that are recognized by autoantibodies in RA patients. The exact mechanism of how oral bacteria contribute to systemic autoimmunity is still not fully clear, but the growing number of studies suggest that both gut and oral microbiomes have an important role in autoimmune disease onset and progression (Alghamdi and Redwan 2022; Potempa et al. 2017).

### 6.3 Molecular Mimicry and Autoimmunity

Molecular mimicry, where microbial peptides look very similar to host self‐antigens, is another important way that the gut microbiota can trigger autoimmunity. Some bacteria, such as *Bacteroides fragilis*, *Prevotella copri*, and *Streptococcus sanguis*, are able to produce peptides that mimic host proteins and cause cross‐reactive immune responses. These microbial peptides can push the immune system to attack self‐antigens, which can contribute to the development of autoimmune diseases, such as RA and MS (C. Zhou et al. 2020; Fehringer and Vogl 2025). Recent studies emphasize how strong the role of molecular mimicry can be in autoimmune disease. They show that bacterial peptides from gut microbes can resemble host antigens and, in this way, activate the immune system. For instance, peptides from Akkermansia muciniphila and Ruminococcus gnavus have been linked to type 1 diabetes because they mimic pancreatic beta‐cell antigens. Similarly, *Klebsiella pneumoniae* has been connected to ankylosing spondylitis due to structural similarities with human leukocyte antigen B27 (HLA‐B27), which is a key marker for this disease (El Maghraby et al. 2023; Maytin and Morrison 2022).

### 6.4 Genetic Factors in Autoimmunity and Microbiome Interactions

Although dysbiosis can change immune responses a lot, genetic factors, especially differences in human leukocyte antigen (HLA) genes, also have a big role in deciding who is more likely to develop autoimmune disease. Certain HLA types, like HLA‐DR2 and HLA‐DR3 in lupus, HLA‐DRB1*04 in RA, and HLA‐DR3 and HLA‐DR4 in type 1 diabetes, are known to increase disease risk. When these genetic backgrounds are combined with imbalances in the gut microbiota, they create a complex interaction that can trigger or make autoimmune responses worse (Klimenta et al. 2019; Pahkuri et al. 2024). The way genetics and the microbiome interact shows that we still need a deeper understanding of how specific microbial communities and genetic predisposition work together in autoimmunity. If we can map this better, it might be possible in the future to design more personalized treatments that target both the microbiome and genetic risk factors, opening new ways to prevent or manage autoimmune diseases (Scher et al. 2025) (Table 2).

**Table 2:** Therapeutic approaches targeting the microbiome in autoimmune diseases.

Therapeutic approach	Description	Impact on autoimmunity	Targeted mechanisms	Examples	
Probiotics	Probiotic therapy, using beneficial microorganisms, can restore gut balance and regulate immune responses.	Probiotics reduce gut inflammation, improve immune function, and prevent autoimmune responses.	Restores microbial balance, regulates Treg/Th17 cell ratio, and reduces inflammatory cytokines.	Probiotic use in treating conditions, like IBD, MS, and RA.	Atabati et al. (2021) and Jia et al. (2020)
Dietary interventions	Increasing fibers and immune‐modulating components (e.g., sweeteners) can positively impact microbiome composition.	Dietary modulation can support immune health, reduce inflammation, and correct dysbiosis.	Promotes beneficial microbes, reduces harmful inflammation, and supports immune tolerance.	Increasing fiber intake to improve gut microbiome and immune regulation.	Fetarayani et al. (2025) and Jank and Bhargava (2024)
Fecal microbiota transplantation (FMT)	FMT involves transferring gut microbiota from healthy individuals to autoimmune patients to restore balance and immune tolerance.	FMT has shown promise in modulating immune responses and alleviating autoimmune disease symptoms.	Restores balance in the gut microbiome, improving immune function and reducing disease progression.	FMT trials in diseases like ulcerative colitis and Crohn's disease.	Bi et al. (2025) and Waller et al. (2022)
Synbiotics	Synbiotics combine prebiotics and probiotics to enhance their synergistic effects on gut health and immune system regulation.	Synbiotics optimize microbial interactions, improving immune homeostasis and managing inflammation.	Enhances synergistic effects of prebiotics and probiotics to control inflammation and regulate immune response.	Synbiotics for improving gut flora balance and managing inflammatory conditions.	Kumari et al. (2025) and Yue et al. (2025)
Postbiotics and metabiotics	Postbiotics, such as short‐chain fatty acids and melatonin, are produced by microbial fermentation and can mitigate inflammation.	Postbiotics can regulate immune signaling pathways, reducing inflammation and promoting immune tolerance.	Produces beneficial metabolites that mitigate systemic inflammation and promote immune regulation.	Postbiotics for controlling gut‐related inflammatory diseases.	Cristina et al. (2025) and P. Zhou et al. (2024)

Abbreviations: IBD, inflammatory bowel disease; MS, multiple sclerosis; RA, rheumatoid arthritis; Th17, T‐helper 17 cell; Treg, regulatory T cell.

### 6.5 Impact of Pharmaceutical Agents on Gut Microbiota and Autoimmune Responses

Disruptions to the microbial ecosystem—referred to as dysbiosis—can alter immune signaling pathways and contribute to the development or progression of autoimmune diseases. Pharmacological agents such as antibiotics and immunosuppressive drugs are among the most significant external factors capable of reshaping gut microbial communities and modulating host immune responses. The broad‐spectrum activity of the antibiotics can disrupt the composition and function of the intestinal microbiota. Antibiotic exposure often reduces microbial diversity and alters the relative abundance of major bacterial phyla, including Firmicutes, Bacteroidetes, and Proteobacteria, thereby disturbing microbial metabolic networks and host–microbe interactions (Patangia et al. 2022). Experimental studies have demonstrated that antibiotic treatment can significantly modify gut microbial composition and metabolic output. For instance, antibiotic‐mediated dysbiosis has been associated with reduced production of SCFAs, particularly acetate and butyrate, which play important roles in maintaining intestinal barrier integrity and regulatory immune responses (Taitz et al. 2025). Reduced SCFA production may impair immune tolerance and favor proinflammatory pathways. Several preclinical studies have also shown that antibiotic‐induced microbial perturbations can influence autoimmune disease outcomes. A systematic review of experimental models reported that antibiotic exposure altered key microbial taxa and immune pathways, including regulatory T‐cell (Treg) responses and anti‐inflammatory cytokine production, such as interleukin‐10 (IL‐10) (Gobbo et al. 2022). Several pathways are involved, including disruption of microbial metabolite production, modulation of T‐helper cell differentiation, and altered antigen exposure. In murine models, early antibiotic exposure resulted in reduced levels of beneficial microbial metabolites and decreased regulatory T‐cell populations, accompanied by increased proinflammatory cytokines, such as IL‐4 and IL‐17 (Kim et al. 2020).

Systematic reviews have shown that several commonly used immunosuppressive agents, including tacrolimus, cyclosporine, mycophenolate mofetil, and corticosteroids, can significantly change the composition of the gut microbiome. Studies in transplant recipients have reported shifts in anaerobic bacterial populations, such as Ruminococcaceae, Lachnospiraceae, and Bacteroides (Gibson et al. 2021). These microbiota alterations affect both drug efficacy and host immune responses. Experimental studies have shown that prolonged glucocorticoid therapy can alter microbial diversity and metabolic activity, including reductions in SCFA‐producing bacteria such as Clostridium species and shifts in metabolic pathways related to phenylalanine and SCFA metabolism (J. Zhang, Feng, et al. 2021).

## 7 Clinical Implications of Microbiome Research in Autoimmunity

**Figure 2:**
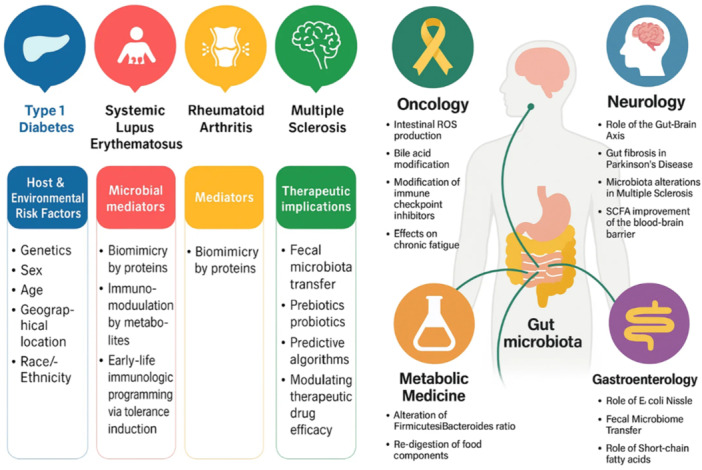
Clinical implications of microbiome research in autoimmunity. ROS, reactive oxygen species; SCFAs, short‐chain fatty acids.

The study of the microbiome has really important meaning for clinical practice, especially for autoimmune diseases. Conditions like MS, lupus, and RA all have the same big problem: we still do not fully understand how they start and most current treatments are not very effective in the long term. Some advanced options, like T‐cell therapy or stem cell therapy, are used in a few countries, but they can be very expensive and also carry serious risks, including sometimes even death (T. Liu et al. 2025; Selmi 2018). Because of this, the idea of probiotic therapy looks like a promising alternative. Probiotics are generally seen as active, “healthy” and relatively cheap options that can support the body instead of just blocking the immune system. They have already shown benefits in things like infections, obesity, and inflammatory bowel disease, with usually only mild side effects (Yu et al. 2025). The idea is that probiotics might help in autoimmune diseases at the cellular level, even before a full immune response is activated, so maybe they could slow down or control the disease in its early stages. As more studies show how strongly the microbiome is connected to autoimmune conditions, the argument for using probiotics in clinical practice becomes stronger (Madras and Larkin 2025). In the future, these microbiome‐based treatments could be written into official clinical guidelines, so doctors would feel more confident to use them as part of routine patient care (Figure 2).

## 8 Emerging Therapeutic Approaches

A main area of interest now is how we can change the gut microbiome on purpose using different diet‐based strategies. This includes things like prebiotics, probiotics, fecal microbiota transplantation, synbiotics, and other nutrition therapies, such as postbiotics and metabiotics. Also, adding nondigestible carbohydrates and polyunsaturated fatty acids to the diet might be another way to influence the microbiome and maybe reduce autoimmune symptoms (Bulut et al. 2025; Smolinska et al. 2025). Some studies show that using a mix (coculture) of beneficial bacteria works better than giving just one strain alone, and can lead to stronger therapeutic effects. Postbiotics like SCFAs, melatonin, and indole derivatives also seem able to reduce inflammation (Yilmaz 2024). For example, melatonin as an extra (adjuvant) therapy together with normal antidepressant drugs has shown some potential to improve depressive symptoms. Small molecules made by certain bacteria can even reverse microbiota imbalances that are linked to obesity and metabolic problems, so they might also help lower depression risk in obese patients (Evrensel and Ceylan 2015). There is also research on banana flakes mixed with lupin that looks promising for regulating the gut microbiome and controlling inflammation in animal models. In general, microbiome‐targeted therapies aim to correct dysbiosis in the gut, bring back a more normal balance of bacteria, and affect immune development in a way that prevents systemic inflammation and related autoimmune conditions (Singh and Bhadauriya 2025). A lot of animal and human studies suggest that microbial bioproducts can improve immune function and help reduce systemic inflammation, which in the end may prevent or control autoimmune responses (Omar et al. 2025; Song et al. 2025). Current clinical evidence indicates that microbiome‐targeted therapies can modulate immune responses in autoimmune diseases, although results remain heterogeneous. Table 3 presents the recent and key clinical studies in practical applications of these interventions in autoimmune diseases.

**Table 3:** Key clinical studies in practical applications of microbiome‐targeting interventions in autoimmune diseases and their outcomes.

Target disease	Study design and population	Strains	Intervention duration	Main outcomes	References
Rheumatoid arthritis	Randomized, double‐blind, placebo‐controlled trial (*n* = 60 RA patients)	*Lactobacillus acidophilus, Lactobacillus casei, Bifidobacterium bifidum*	8 weeks	Significantly reduced Disease Activity Score‐28 (DAS‐28), insulin levels, and CRP	Zamani et al. (2016)
Rheumatoid arthritis	Randomized double‐blind clinical trial in women with RA	*L. casei* 01	8 weeks	Significant reductions in inflammatory biomarkers (CRP and IL‐6) and improved clinical disease activity scores (DAS‐28)	Alipour et al. (2014)
Rheumatoid arthritis	Randomized placebo‐controlled trial in newly diagnosed RA (*n* = 100)	*L. casei, Lactobacillus salivarius, Bifidobacterium breve* (adjunct to DMARD therapy)	12 months	Reductions in disease activity, inflammatory markers (CRP and ESR) and pain scores, higher remission rates	Barac et al. (2025)
Rheumatoid arthritis	Randomized pilot clinical trial	*Lactobacillus rhamnosus* GG	12 months	Probiotic therapy was safe but showed limited effects on overall RA disease activity, highlighting variability in probiotic responses	Hatakka et al. (2003)
Type 1 diabetes mellitus	Randomized placebo‐controlled trial in children/adolescents (*n* = 70)	*L. acidophilus*	6 months	Significant improvements in fasting glucose, HbA1c, lipid profiles, and cytokines (decreased IL‐21 and increased IL‐22), suggesting immunomodulatory effects	Adly et al. (2025)
Type 1 diabetes mellitus	Randomized double‐blind placebo‐controlled trial in children with new‐onset T1D	Multi‐strain De Simone formulation (*Lactobacillus, Bifidobacterium, Streptococcus* species)	3 months	Significant reduction in HbA1c and insulin requirements compared with placebo, improved glycemic control and immune modulation	Kumar et al. (2021)
Type 1 diabetes mellitus	Randomized controlled trial in newly diagnosed pediatric patients	*L. rhamnosus* GG and *Bifidobacterium lactis* Bb12	6 months intervention, 12 months follow‐up	No significant improvement in β‐cell function (C‐peptide levels), illustrating strain‐specific variability	Groele et al. (2021)
Ulcerative colitis (IBD)	Randomized clinical trial comparing probiotic, prebiotic, and synbiotic therapy (*n* = 120 UC patients)	*Bifidobacterium longum*	4 weeks	Improvements in inflammatory bowel disease quality‐of‐life scores, with synbiotic therapy showing greater benefit than probiotics alone	Fujimori et al. (2009)

Abbreviations: CRP, C‐reactive protein; DMARD, disease‐modifying anti‐rheumatic drug; ESR, erythrocyte sedimentation rate; HbA1c, hemoglobin A1c; IBD, inflammatory bowel disease; IL, interleukin; RA, rheumatoid arthritis; T1D, type 1 diabetes; UC, ulcerative colitis.

Autoimmune diseases are becoming a bigger public health problem, especially in Western countries. Studying how genes, diet, lifestyle and the microbiome are connected is very important to find the mechanisms that trigger these diseases (Wuni and Vimaleswaran 2024). To really understand the potential of microbiome‐based therapies, future work should not only look at the presence of microbes, but also at the immune phenotype and the long‐term effects of giving probiotics. Longitudinal studies (following people over time) will be needed to see how long these effects last and to identify which microbiome‐related treatments are actually the most useful for autoimmune diseases (Chuang et al. 2024).

## 9 Adverse Effects and Contraindications

Although generally considered safe in healthy populations, these interventions require careful considerations in patients with autoimmune diseases, primarily due to these concurrent immunosuppressive therapies and altered immune function (Van den Nieuwboer et al. 2015). Several potential adverse effects and contraindications have been reported in clinical and observational studies. The most frequently reported adverse effects are mild GI symptoms, including bloating, abdominal discomfort, and flatulence (X. Liu et al. 2024). These symptoms are generally transient and resolve after continued use or dose adjustment. Overall, most clinical trials report that probiotics are well tolerated, although reporting of adverse events in probiotic studies remains inconsistent. A systematic evaluation of clinical studies involving immunocompromised adults found that probiotic and synbiotic supplementation was generally safe and well tolerated; however, the authors noted substantial variability in study design and adverse event reporting, highlighting the need for standardized safety monitoring in probiotic research (Van den Nieuwboer et al. 2015).

Although rare, systemic infections associated with probiotic organisms have also been documented (Yelin et al. 2019). These include bacteremia, fungemia, and sepsis caused by microorganisms commonly used in probiotic formulations, such as *Lactobacillus, Bifidobacterium, Bacillus*, and *Saccharomyces* species. Such events are most frequently reported in immunocompromised individuals, critically ill patients, or those with impaired intestinal barrier function. Genomic analyses confirmed that *Lactobacillus* strains isolated from blood cultures in intensive care unit patients were genetically identical to strains present in administered probiotic products, providing direct evidence that probiotic organisms can occasionally cause bacteremia. Similarly, there are cases reporting *Lactobacillus* bacteremia associated with probiotic use in pediatric hematopoietic stem cell transplant recipients, emphasizing the potential risks in severely immunocompromised populations (Gilliam et al. 2023). Additional reports have documented bloodstream infections caused by probiotic organisms, such as *Bacillus clausii* and *Bacillus licheniformis*, following probiotic administration in hospitalized or critically ill patients (Corredor‐Rengifo et al. 2024; Zou et al. 2024).

Although formal contraindications are not universally established, several patient groups are considered higher risk for probiotic‐associated complications. These include individuals who are severely immunocompromised (including those receiving chemotherapy or high‐dose immunosuppressive therapy), critically ill or hospitalized (particularly admitted to ICUs), patients with central venous catheters or indwelling medical devices, and individuals with severely impaired intestinal barrier function, such as severe mucosal inflammation or short bowel syndrome (Snydman 2008; Katkowska et al. 2021). In these populations, the risk of microbial translocation and opportunistic infection may be increased, and probiotic use should therefore be approached cautiously and ideally under medical supervision.

## 10 Challenges and Limitations

Despite growing interest in microbiome‐targeted therapies, their clinical application in autoimmune diseases remains limited by several important scientific, clinical, and regulatory challenges.

### 10.1 Interindividual Variability in Microbiome Composition

One of the primary obstacles to microbiome‐directed therapies is the substantial interindividual variability in baseline gut microbiota composition. The human intestinal microbiome is affected by various factors, including host genetics, diet, age, medication use, geographic environment, and disease state. Thus, individuals often respond differently to the same microbiota‐modulating intervention. Evidence suggests that the ability of administered probiotic strains to colonize the intestine and exert immunomodulatory effects is highly dependent on the host's existing microbial ecosystem. In some individuals, probiotics may transiently colonize the gut, whereas in others they may fail to engraft due to competition with established microbial communities. This variability contributes to inconsistent clinical outcomes observed across trials evaluating microbiome‐based therapies (Durack and Lynch 2019).

### 10.2 Potential Adverse Effects and Safety Concerns

Although probiotics are generally considered safe for the majority of the population, adverse effects have been reported, particularly in vulnerable patient groups. Mild GI symptoms, such as bloating, flatulence, and abdominal discomfort, are among the most commonly reported side effects (X. Liu et al. 2024). More rarely, serious complications such as bacteremia, fungemia, or sepsis caused by probiotic organisms have been documented, especially in immunocompromised individuals, critically ill patients, and those with central venous catheters or compromised intestinal barriers (X. Liu et al. 2024). Additionally, interactions between gut microbes and host metabolism may influence drug metabolism and immune signaling pathways. The emerging field of pharmacomicrobiomics demonstrates that gut microorganisms can modify the metabolism and bioavailability of certain medications, potentially affecting therapeutic efficacy or adverse event profiles (Bolte et al. 2025).

### 10.3 Challenges in Achieving Consistent Therapeutic Efficacy

Another significant limitation is the difficulty in achieving reliable and sustained therapeutic efficacy. Many probiotic strains do not permanently colonize the GI tract, and their effects may be transient (Han et al. 2021). Moreover, the immunomodulatory properties of probiotics are often strain‐specific, meaning that results obtained with one strain cannot be generalized to others, even within the same species. Variability in dosage, formulation, treatment duration, and host factors further complicates the interpretation of clinical trial results. Consequently, despite promising experimental data, large‐scale clinical trials often produce heterogeneous outcomes, limiting the development of standardized therapeutic protocols for autoimmune diseases.

### 10.4 Regulatory and Standardization Challenges

In many countries, probiotics are classified as dietary supplements rather than pharmaceutical products, resulting in less stringent requirements for clinical efficacy and quality control. This regulatory variability contributes to significant differences in product composition, microbial viability, labeling accuracy, and manufacturing practices (Spacova et al. 2023). Furthermore, standardization of probiotic formulations and large‐scale production presents additional challenges. Maintaining microbial viability during manufacturing, storage, and distribution is technically demanding, and strain‐specific characteristics must be preserved to ensure biological activity (Grumet et al. 2020).

## 11 Conclusion

The link between the gut microbiome and autoimmune diseases is quite complex and probably goes in both directions. Research shows that the human microbiome is shaped by many different factors, like genetics, diet, lifestyle, and environment (e.g., living with pets or in different regions of the world). Medicines, especially antibiotics and some immunosuppressive drugs, also have a strong effect on the microbiota. At the same time, it is well known that diet and environmental conditions can change both the microbiome and the immune system, influencing whether a person stays healthy or develops disease. On the other side of this interaction, commensal microbes living in the gut play a key role in immune development and long‐term maintenance. They support the expression of innate pattern‐recognition receptors, help with the recruitment of immunosuppressive cells like regulatory T cells (Tregs), and give protection against infections.

In people with autoimmune diseases, their microbiota may be more fragile or “at risk,” which can increase the chance of disease development or relapse. Animal studies have shown that gut microbiota affects not only how many Tregs there are and how they function, but also their differentiation and programming. The presence of Tregs in the thymus of germ‐free mice suggests that the microbiome may also take part in Treg generation inside the thymus itself. There is even the possibility that some bacteria or other microbes from the gut might move outside the intestine and cause abnormal Treg responses in genetically predisposed hosts. Because Tregs are so important for keeping autoimmunity under control, one idea is that the gut microbiome, by interacting with these cells, can influence if and how autoimmune diseases appear. However, these hypotheses are still under investigation, and other explanations may also exist.

Most of the current research on the microbiome and autoimmunity has been done in animal models. The data are promising, but there are limits in how valid and consistent the studies are. Many experiments use small sample sizes and different methods, which leads to scattered and sometimes conflicting results, so it is hard to get very clear conclusions. Human studies have similar problems, like bias and large variability between individuals. Still, animal models have the advantage that researchers can control the environment and follow disease induction and resolution in a more systematic way. Right now, we still do not know for sure whether specific microbiome manipulations can give strong and lasting clinical benefits in humans, but it is already clear that the microbiome has an important role in autoimmunity. More robust and larger studies, including meta‐analyses, are needed to better understand the mechanisms and to improve therapeutic strategies. As research continues, changing or “tuning” the microbiome may help reduce inflammation, delay the onset of autoimmune diseases, and open new treatment options. So, the available evidence supports the idea that the microbiome is a real factor in autoimmunity, and future therapies might use modulation of gut microbiota as part of effective management of autoimmune diseases.

## Author Contributions

**Behshad Boroumand:** data curation, conceptualization, methodology. **Ghazal Zamani:** writing – original draft, writing – review and editing. **Ehsan Zandi:** writing – review and editing, writing – original draft. **Milad Vahedinezhad:** writing – review and editing, writing – original draft. **Elham Abdollahi:** writing – original draft, writing – review and editing. **Sepideh KarkonShayan:** writing – review and editing, writing – original draft. **Mustafa Sattar:** writing – review and editing, writing – original draft.

## Funding

The authors have nothing to report.

## Ethics Statement

The authors have nothing to report.

## Consent

The authors have nothing to report.

## Conflicts of Interest

The authors declare no conflicts of interest.

## Data Availability

The authors have nothing to report.
